# Transcriptional effects of a positive feedback circuit in *Drosophila melanogaster*

**DOI:** 10.1186/s12864-017-4385-z

**Published:** 2017-12-28

**Authors:** Jarosław Bryk, R. Guy Reeves, Floyd A. Reed, Jai A. Denton

**Affiliations:** 1 Department of Evolutionary Genetics, Max Planck Institute for Evolutionary Biology, Plön, Germany; 2 School of Applied Sciences, University of Huddersfield, Huddersfield, HD1 3DH UK; 3 Department of Biology, University of Hawaiʻi at Mānoa, Hawaiʻi, Honolulu, 96822 USA; 4 Genomics & Regulatory Systems Unit, Okinawa Institute for Science & Technology Graduate University, Onna-son, Okinawa, Japan

**Keywords:** *Drosophila melanogaster*, tTAV, Vector control, Transcriptome, Microarrays, Tetracycline

## Abstract

**Background:**

Synthetic systems that use positive feedback have been developed to control human disease vectors and crop pests. The tTAV system, which has been deployed in several insect species, relies on a positive feedback circuit that can be inhibited via dietary tetracycline. Although insects carrying tTAV fail to survive until adulthood in the absence of tetracycline, the exact reason for its lethality, as well as the transcriptomic effects of an active positive feedback circuit, remain unknown.

**Results:**

We engineered the tTAV system in *Drosophila melanogaster* and investigated the effects of tTAV genome integration locus on the whole fly transcriptome during larval and adult life stages in four transgenic fly strains using gene expression microarrays. We found that while there were widespread effects on the transcriptome, the gene expression differences after removal of tetracycline were not consistent between integration sites. No specific region of the genome was affected, no common set of genes or pathways, nor did the integration site affect the transcripts in *cis*.

**Conclusion:**

Although the positive feedback tTAV system is effective at killing insect larvae regardless of where it is inserted in the genome, it does not exhibit a specific, consistent transcriptional signature. Instead, each insertion site is associated with broad, but different, transcriptional effects. Our results suggest that lethality may not be caused by a direct effect on transcription of a set of key genes or pathways. Instead, we propose that rather than a specific action of a tTAV protein, it is the stochastic transcriptional effects specific to each insertion site that contribute to the tTAV-induced mortality.

**Electronic supplementary material:**

The online version of this article (10.1186/s12864-017-4385-z) contains supplementary material, which is available to authorized users.

## Background

Synthetic gene circuits that rely on positive feedback have been developed to replace irradiation-based methods for sterile insect technique (SIT) [1–4]. SIT has been an effective control strategy for insect populations, for more than 60 years, where sterilized males of a particular species are released and mate with females from the target population [5, 6]. It is species-specific, environmentally friendly, and has been effective at controlling a wide variety of insects. Molecular techniques can potentially improve the efficiency and effectiveness of SIT through the development of simple synthetic gene circuits that confer lethality or allow selection of only male insects for release [1, 2, 4, 7, 8].

A synthetic system that relies on a positive feedback gene circuit has been deployed in the mosquito *Aedes aegypti* [1, 2, 9–11]. This system, called OXI513a, has moved beyond field trials and has been used to suppress targeted mosquito populations [9, 12]. Like all SIT systems, population suppression is achieved via the release of OX513a male mosquitoes that mate with local females to produce no or very few offspring that survive to adulthood.

**Fig. 1:**
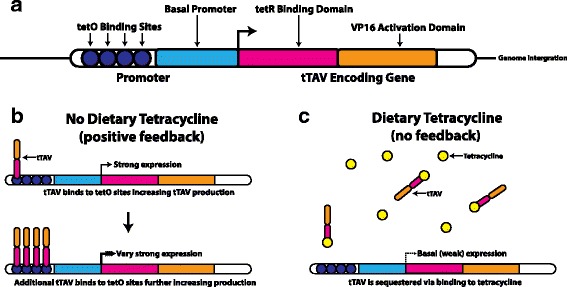
The TransActiVator (tTAV) feedback circuit. **a** Schematic of the tTAV system. **b** In the absence of tetracycline, basal levels of tTAV protein bind its own promoter at the TetO sites, thereby increasing tTAV protein production. This new protein binds the promoter and further increases expression. **c** In the presence of tetracycline, low levels of tTAV are sequestered and expression of tTAV mRNA remains at basal levels

The lethality system used in OXI513a relies on the product of a single gene, tetracycline-controlled TransActiVator (tTAV), which enhances its own expression [1–3]. Positive feedback is generated via basal expression of the tTAV activator protein, a fusion of the tetracycline-binding domain (tetR) and the herpes simplex virus transcriptional activator (VP16) [13]. In the absence of tetracycline, the tetR domain of the tTAV protein binds to one of several TetO sequences upstream from the tTAV-encoding region. Each binding of tTAV further increases its own expression, via its VP16 domain. However, when an organism is provided with sufficient dietary tetracycline, which functions as an inhibitor, the tetR domain binds the antibiotic. The feedback is suppressed and the expression of tTAV remains at the low levels established by the basal promoter (Fig. 1). The suppression means the dominant lethality is inhibited and the insects can be produced in large numbers for release.

In the absence of tetracycline, activation of the tTAV positive feedback system kills insects before they become reproductive, making this an effective control system in a post-release environment. However, unlike other genetic tTAV based systems that rely on control of gene expression induced lethality [4, 7, 14, 15], the exact reason for lethality of the positive feedback system remains unknown. Proposed mechanisms include tTAV toxicity, change in expression of critical transcripts, transcriptional squelching, wherein localised transcriptional machinery is titrated away, or overloading of the ubiquitin protease pathway due to high protein production [1, 3]. Complicating the understanding of this phenomenon is a very limited knowledge on the effects of synthetic positive feedback circuits on an organism’s transcriptome.

The tTAV system has been introduced into several insect species, but in some insertion sites the tTAV system is recessive lethal, even when not active, and therefore cannot be homozygous. A strain homozygous for the tTAV system was reported in *A. aegypti* and *Ceratitis capitata* (medfly) but not *D. melanogaster* [1, 3]. Moreover, a similar tTAV system that included an intron and multiple tTAV encoding regions, when introduced into *Pectinophora gossypiella* (pink bollworm) was predominantly recessive lethal, complicating the generation of a homozygous line [16].

The tTAV positive feedback systems function as expected in each insect species tested, in that tTAV expression is repressible with dietary tetracycline and lethal without it. However, the timing of lethality varied among species and even between insertion sites within the same species. In *P. gossypiella*, death reportedly occurred in the larval stage [16]. Whereas, of the three *A. aegypti* lines that displayed tetracycline repressible dominant lethality, two died in early larval stages, and the third died during the transition from late larvae to early pupae [2]. This insertion site-specific variation in the lethality timing was also noted in *C. capitata* [3].

Compared to animals grown on dietary tetracycline, tTAV expression is enhanced 36-fold in *C. capitata* third instar larvae and 48-fold in adults deprived of tetracycline for 4 days [3]. *A. aegypti* adults removed from 100 μg/mL tetracycline had a 150-fold increase tTAV expression after 4 days [1]. Finally, *D. melanogaster* adults had 46-fold and 69-fold increases after 1 and 4 days, respectively, after deprivation of 100 μg/mL tetracycline [1]. This highlights the responsiveness of the tTAV system, showing that the suppressive effect of residual tetracycline is quickly overcome. In each of these three species no impact on adult lifespan or health could be detected after removal of dietary tetracycline [1, 3]. Also, when adult mice carrying a tTAV-based system were no longer provided dietary tetracycline they lived for six months without observable ill effects [17]. It was proposed that this lack of lethality in adults may reflect transcriptional squelching or that interruptions to ubiquitin-dependent protein degradation may be less harmful to adults [3]. It may also be that the tTAV feedback circuit elicits a diminished response, at the transcriptomic level, in adults.

Understanding what factors contribute to the variance in tTAV-associated mortality is likely to inform the development of insect control systems. In the present study, the OXI513a tTAV feedback circuit was introduced and verified into *D. melanogaster* [1]. The tight tetracycline regulation of the system affords a convenient method to conduct a strain-by-strain assessment of the tTAV system and determine the transcriptomic effect, if any, of a positive feedback circuit. Transcriptomic analysis of four independent *D. melanogaster* tTAV insertion lines, in both adults and larvae, was conducted to examine the transcriptomic influence of the tTAV system.

## Results

### The tTAV lethality system in *D. melanogaster*

A tTAV positive feedback circuit, based on the LA513 plasmid, used in the development of *A. aegypti* OXI513a, [1], was developed for use in *D. melanogaster*. Using PhiC31 landing sites the above plasmid was integrated at specific widely used locations throughout the *D. melanogaster* genome (Table 1 and Methods). These landing sites represent a range of genomic locations that include each of the *D. melanogaster* chromosomes except the Y. Moreover, the chosen strains contain very well characterised homozygous landing sites. The tTAV heterozygous (technically hemizygous, but the term heterozygous is used here for convenience) stocks contained balancer chromosomes and, although viable when provided dietary tetracycline, failed to produce adult transgenic offspring in the absence of tetracycline. Lethality usually occurred prior to the third instar stage of larval development. Specifically, the onset of the lethality phenotype commenced part way through the second instar. Generally, all larvae would die prior the third instar but very rarely single third instar larvae would crawl up the side of control vials; none of these individuals would pupate prior to dying. Unlike tTAV in other organisms, not obvious difference in lethality timing between insertion sites was detected. As in previous tTAV systems, adults could be deprived of tetracycline without any observed ill effects. Of 10 integration loci tested, nine strains failed to produce homozygous offspring when raised on 100 μg/mL dietary tetracycline. Only cytological position 102D was viable as a homozygote. Increasing dietary tetracycline dosage to 200 μg/mL did not permit tTAV homozygosity or the establishment of a strain containing two tTAV integrations at both, adjacent, cytological positions 51C and 51D. However, crosses with 100 μg/mL dietary tetracycline between strains containing the tTAV integration at position 51C, on the second chromosome, and 76A2 or 86Fb, on the third chromosome, produced offspring containing both two tTAV integrations. Furthermore, an insertion at locus 19E7, on the X chromosome, produced viable male flies.

**Table 1:** Details of tTAV stocks, insertion sites and viability. Viability is the ability to survive when grown on media containing 100 μg/mL tetracycline. The tTAV hemizygous stocks contained balancer chromosomes. All tTAV flies die in the absence of tetracycline

Chromosome – Cytological Site	Viability
X – 19E7	Hemizygous Viable / Male Viable / Homozygous Lethal
2 L – 22A	Hemizygous Viable / Homozygous Lethal
2 L – 28E7	Hemizygous Viable / Homozygous Lethal
2R – 43A1	Hemizygous Viable / Homozygous Lethal
2R – 51C	Hemizygous Viable / Homozygous Lethal
2R – 51D	Hemizygous Viable / Homozygous Lethal
3 L – 68E	Hemizygous Viable / Homozygous Lethal
3 L – 76A2	Hemizygous Viable / Homozygous Lethal
3R – 86Fb	Hemizygous Viable / Homozygous Lethal
4 – 102D	Hemizygous Viable / Homozygous Viable

To ensure that the tTAV circuit behaved consistently, with respect to the previous systems, a series of survival tests were conducted. A two-generation crossing regime was used, wherein tTAV-102D males were crossed with ‘white eyed’ (*w* ^*1118*^) females, and their offspring were backcrossed to the same *w* ^1118^ line (Table 6 and Methods).

**Fig. 2:**
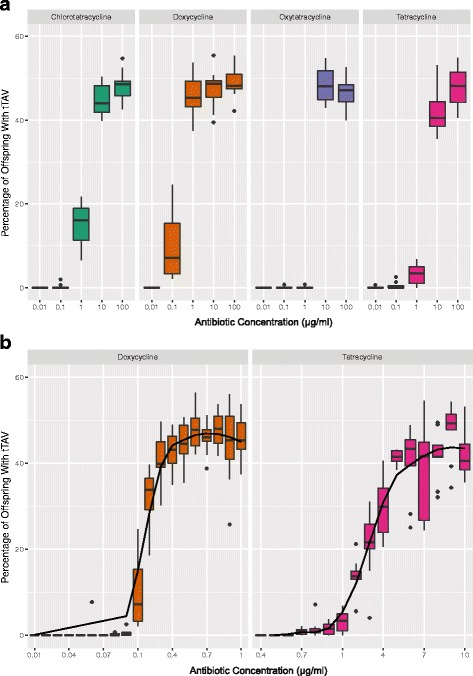
Characteristics of the tTAV circuit. The crossing scheme and media used are described in detail in the Methods, but, briefly, the homozygous 102D–tTAV line was crossed to a ‘white eyed’ w1118 line. Red-eyed wildtype flies, indicating the presence of the tTAV-system, were back crossed to the w1118 line and the percentage of tTAV flies in the F2 generation on various types and concentrations of antibiotic media was determined. Due to the use of tTAV heterozygotes, 50% survival represents the maximum percentage of the offspring that can inherit tTAV. Each data point is the median of 10 replicates. The data are presented as a standard boxplot with whiskers extending to the lowest or highest value within 1.5 times the IQR from the hinge. **a** Median survivorship for 4 members of the tetracycline class of antibiotics across dosages spanning 5 orders of magnitude. **b** Higher dosage resolution of median survivability for tetracycline and doxycycline, showing critical thresholds for antibiotic rescue. The IC50 values for doxycycline and tetracycline are 0.161 and 3.338, respectively

Dietary concentrations of tetracycline, doxycycline, oxytetracycline, or chlorotetracycline varying over five orders of magnitude (0.01 to 100 μg/mL) were tested for their ability to rescue tTAV-102D in the aforementioned two-generation cross (Fig. 2a). Survival shows a clear response to dietary tetracycline concentration, with doxycycline capable of rescuing tTAV system lethality at a concentration almost an order of magnitude lower than other tetracycline homologs.

Both tetracycline- and doxycycline-induced survival had a similar, almost logarithmic dose response, despite the 10× difference in dosage. A more exact determination of the rescue threshold for tetracycline and doxycycline was obtained by repeating the above experiment with increased tetracycline dosage resolution (Fig. 2b). Tetracycline demonstrated a sharp decline in its capacity to rescue tTAV lethality between 5 μg/mL and 1 μg/mL whereas doxycycline demonstrated a similarity sharp decline between 0.4 μg/mL and 0.1 μg/mL.

### Microarray analyses

To investigate the influence of the tTAV feedback circuit on gene activity, we assessed the changes in the transcriptome with and without tetracycline. This system provides an ideal internal control when examining flies raised with versus without tetracycline, as it allows us to differentiate transcriptional effects of the tTAV system in different genetic backgrounds and life stages (see Fig. 6 for the experimental design). We initially examined the transcriptional effects with and without tetracycline in a homozygous strain 102D–tTAV, but as we were unable to generate additional viable homozygous strains, we subsequently analysed three additional chromosome-balanced heterozygous tTAV strains. We also analysed a non-tTAV line. In each of the lines, we analysed two life stages, adults and second instar larvae, every time comparing gene activity with versus without tetracycline for each strain and life stage (Table 2). Due to the use of balancer chromosomes, wherein balancer homozygotes die as embryos, and the tTAV recessive lethality at these loci, the genotype of all offspring is known without the need for phenotypic selection.

**Table 2:** Fly Stocks used for Microarray

Strain ID	Genotype	Bloomington ID	Genome Integration
non-tTAV	y^1^; Gr22b^iso-1^ Gr22d^iso-1^ cn^1^ CG33964^iso-1^ bw^1^ sp^1^; LysC^iso-1^ MstProx^iso-1^ GstD5^iso-1^ Rh6^1^	2057	
51D–tTAV	y^1^ M{vas-int.Dm}ZH-2A w^a^; M{3xP3-RFP.attP, *w[+mc],* tTAV}ZH-51D/CyO	24483^a^	Dmel_2R-15,054,298
76A2 - tTAV	y^1^ w^1118^; PBac{y + -attP-9A, *w[+mc],* tTAV}VK00013/TM1	9732^a^	Dmel_3L-19,204,358
86F - tTAV	y^1^ M{vas-int.Dm}ZH-2A w^a^; M{3xP3-RFP.attP, *w[+mc],* tTAV}ZH-86Fb/TM1	24749^a^	Dmel_3R-11,808,607
102D - tTAV	y^1^ M{vas-int.Dm}ZH-2A w^a^; M{3xP3-RFP.attP, *w[+mc],* tTAV}ZH-102D	24488^a^	Dmel_4–988,349

^a^denotes progenitor strain ID

### Expressed genes are shared among strains and within developmental stages

**Fig. 3:**
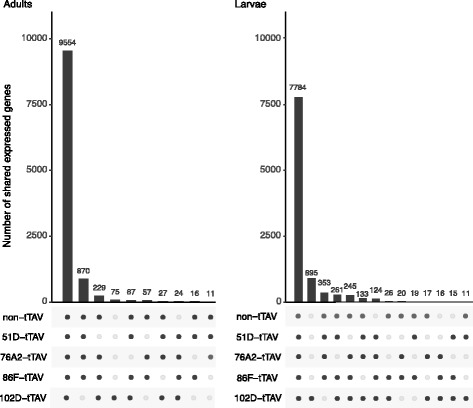
Number of shared expressed genes in all strains for adult and larval flies. Bar height represents the number of expressed genes and the black dots below indicate to which of the lines the genes belong. Sets with fewer than 10 genes are not shown. In both stages most of the genes are expressed in all five lines

Overall, 10,950 genes were expressed in adults and 9947 in larvae, across all strains and treatments (Methods). Most expressed genes were shared among all strains in each developmental stage: 9554 genes of the 10,950 expressed in adults (87%) were expressed in all adult strains and treatments and 7784 of 9947 genes expressed in larvae (81%) were shared among all larvae strains and treatments (Table 3 and Fig. 3).

**Table 3:** Number of expressed genes in each strain and stage

	non-tTAV	102D–tTAV	76A2-tTAV	86F–tTAV	51D–tTAV
Adult Flies	10,844	9986	10,784	10,831	10,575
Larval Flies	8881	9536	8715	8875	8720

### Differentially expressed genes are not shared among different tTAV strains

We next analysed differential gene expression with and without tetracycline within each strain and developmental stage. We performed a moderated t-test for differential gene expression and set the false discovery rate (FDR) at 10% to identify differentially expressed genes (Methods).

At this cut-off, there were no genes differentially expressed due to dietary tetracycline in either larvae or adults in the non-tTAV control. Homozygous 102D–tTAV adults, however, stood out, with 2301 differentially expressed genes compared to 0, 1, and 3 in heterozygous adult tTAV strains. In contrast, in larvae, heterozygous strain 76A2-tTAV had 2116 differentially expressed genes compared to 115, 311, and 336 in the other larval strains (Table 4).

**Table 4:** Number of differentially expressed genes in each strain and life stage at FDR = 10%

	non-tTAV	102D–tTAV	76A2-tTAV	86F–tTAV	51D–tTAV
Adult Flies	0	2301	0	3	1
Larval Flies	0	311	2116	115	336

**Fig. 4:**
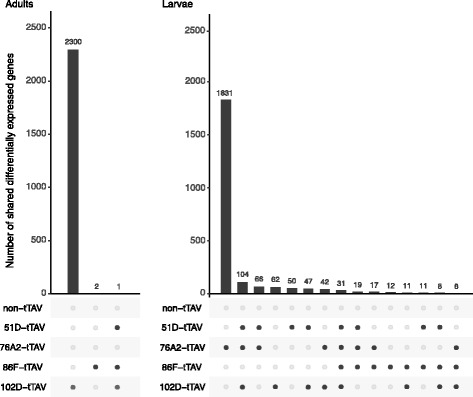
Number of shared differentially expressed genes in all transgenic strains for adult and larval flies. Bar heights represents the number of differentially expressed genes and black dots below indicate to which of lines genes belong. In both stages most of the differentially expressed genes are only present in a single line

Crucially, we detected little overlap in differentially expressed genes with vs. without tetracycline in any transgenic strains or life stages. Among adult tTAV flies, there were no shared differentially expressed genes. Among tTAV larvae, however, there were 31 differentially expressed genes common to all strains (Fig. 4).

When we investigated these 31 genes more closely, we found that 27 of them had opposite directions of expression change depending on the strain (in some strains a gene had higher expression without tetracycline and in other strains expression was higher with it). Only four genes showed a consistent pattern of expression change across all transgenic larval strains: *crok* (CG17218, FBgn0032421) had higher expression with tetracycline (i.e with tTAV system off), whereas *Cyp6a17* (CG10241, FBgn0015714), *olf186-F* (CG11430, FBgn0041585), and *Pex23* (CG32226, FBgn0052226) had higher expression without tetracycline (i.e. with tTAV system on). However, while these differences were statistically significant, they were very small: the range of fold changes in expression level with vs. without tetracycline in all larvae strains was between 0.72 and 1.74. Table 5 shows fold changes of expression levels and adjusted *p*-values for each of the four genes.

**Table 5:** Shared differentially expressed genes show very small differences with versus without tetracycline. Fold change of expression levels and adjusted p-values for each of the four genes that showed consistent expression level change with versus without tetracycline in each transgenic larvae strains. Fold change is higher than 1 if expression increased off of tetracycline

	*Crok*	*Cyp6a17*	*olf186-F*	*Pex23*
102D–tTAV				
Fold Change	0.81	1.34	1.10	1.03
adj p-value	0.05	0.05	0.03	0.02
76A2-tTAV				
Fold Change	0.97	1.33	1.05	1.24
adj p-value	0.01	0.01	0.01	<0.01
86F–tTAV				
Fold Change	0.72	1.03	1.18	1.05
adj p-value	0.08	0.08	0.05	0.02
51D–tTAV				
Fold Change	0.91	1.74	1.14	1.04
adj p-value	0.03	0.04	0.01	<0.01

### Differentially expressed genes in different strains do not belong to the same functional categories

We next investigated whether differentially expressed genes in each line and developmental stage were over-represented in similar functional categories. We observed very little overlap between compared groups, with gene ontology (GO) categories of fatty acid metabolism (GO:0006631, GO:0006635 and GO:0009062), C-acyltransferase activity (GO:0016408), protein deubiquitination (GO:0016579) and cellular lipid catabolic processes (GO:0044242) occurring in larvae 102D–tTAV and 51D–tTAV and mitochondrial part (GO:0044429) being common to 102D–tTAV adults and 76A2-tTAV larvae (Additional File 1: Table S1). We also analysed GO categories for the 31 genes that were differentially expressed in all four transgenic larvae strains. Among 14 categories found, three were related to ubiquitin-specific protease (GO:0004843) and to ubiquitinyl hydrolase activity (GO:0036459 and GO:0101005) (Additional File 2: Table S2).

### Differences in gene expression are not correlated between strains

**Fig. 5:**
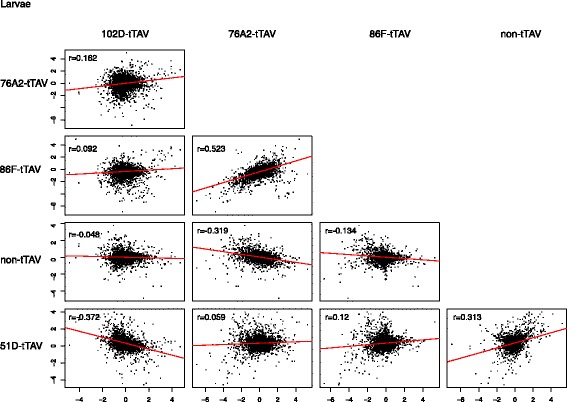
Genome-wide correlations between differences in expression levels with versus without tetracycline for each pair of tTAV larval strains. r is Pearson’s correlation coefficient. All correlations are statistically significant with the largest, *p* < 2 × 10–5

Given the limited consistency in the response to tetracycline treatment in various strains, we checked whether the gene expression differences with vs. without tetracycline in each strain might be correlated. Such correlation would indicate a consistent change in gene expression levels across many genes in each strain, irrespective of the significance of a small number of individual genes. We calculated all pairwise Pearson’s correlation coefficients of differences between expression levels with versus without tetracycline in each strain. We found that while all genome-wide correlations were statistically significant, including correlations with the non-tTAV strain (the largest *p* < 2 × 10^−5^), the sign of the correlation varied (Fig. 5).

### tTAV genomic insertion locus does not affect transcription of nearby genes

Given that we observed virtually no shared differentially expressed genes, no consistent functional categories of differentially expressed genes, nor correlations of gene expression differences with versus without tetracycline in different lines and life stages (Figs. 3, 4 and 5), we investigated whether the physical location of the transgene could explain these observations. As each of the strains had tTAV inserted in a different genomic location, tTAV transgene affecting genes in its physical proximity could be responsible for the observed pattern.

We tested for such cis-acting effects by analysing the distribution of differentially expressed genes in the genome with a sliding window approach. We constructed overlapping gene windows containing 10 genes each and overlapping a neighbouring window by 9 genes, thus ensuring single-gene resolution of the potential effects. We then counted windows in which the proportion of differentially expressed genes was above an arbitrarily chosen threshold of 30%. We observed no clear relationship between the insertion loci and location of differentially expressed genes. There were no windows with more than 3 differentially expressed genes in any strain or life stage, except in 102D–tTAV adults and 76A2-tTAV larvae, where 202 (out of 9529, 2%) and 387 (out of 7764, 5%) windows, respectively, showed more than 3 differentially expressed genes, consistent with the very large number of differentially expressed genes in these strains and life stages (Additional File 3: Figure S3 and Fig. 4).

To test whether the observed distribution of windows was statistically significant, we performed a permutation test, in which we randomly placed the genes along each chromosome or chromosome arm and re-calculated the number of windows in which the proportion of differentially expressed genes was above 30%, for 10,000 iterations. The only significant value we observed for these comparisons was for chromosome 2 L in larval strain 76A2-tTAV (*p* = 0.0338), potentially indicating that the distribution of 10-gene windows with more than 3 differentially expressed genes on chromosome 2 L in this strain and developmental stage was non-random. We note, however, that in this strain the transgene is located on chromosome 2R.

## Discussion

Use of increasingly complex gene circuits for biotechnology and research applications necessitates understanding their influences on the systems in which they are placed. To this end, development of any genetic circuit, such as the tTAV system, in a well-characterized multicellular model organism like *D. melanogaster*, allows assessment of transcriptomic perturbations.

Although we detected insertion site variation, we found no large differences between insertion sites in timing of lethality, unlike those described in the *A. aegypti* or *C. capitata* [1–3]. A possible explanation for the reduced insertion site variation is that tTAV systems employ a *D. melanogaster* basal promoter. Although the use of heterologous promoters is routine, particularly in non-model organism transgenic work, significant genomic differences exist due to the large evolutionary distance between *D. melanogaster* and *A. aegypti* [18–20]. These differences may lead to less predictable transcription behaviour.

The tTAV system reported here responds to tetracycline analogues in a dose-dependent fashion (Fig. 2a & b) and doxycycline rescues tTAV lethality at a concentration almost 10 times lower than tetracycline (Fig. 2b). These results are very similar to those from other systems [4, 21], as expected given the behaviour of doxycycline in commercial TET systems, such as the Clontech TET-On system. The integration-site-specific recessive lethality of the tTAV system, even when inactivated via dietary tetracycline, coupled with the viable males with X chromosome integration and the viability of double heterozygotes, suggests an interaction more complex than simple dosage of the tTAV protein. Moreover, as double heterozygotes of close insertion sites, 51C and 51D, were also non-viable, a possible explanation is that the tTAV system is subject to transvection effects on gene expression [22]. However, extensive further testing, such as placing the tTAV promoter region and the coding region on separate chromosomes, would need to be conducted to confirm this. The timing of lethality to late larvae/early pupae implies disruptions of imaginal disc development [23, 24] or neurogenesis and behaviour [25]. Further elucidation of mechanisms involved in site-specific transcriptional effects might be achieved by quantifying tissue specificities of changes in gene expression in response to positive feedback (e.g., imaginal disc versus mushroom bodies).

To assess the impact of the tTAV system on the transcriptome, and to determine the cause of lethality, we mediated the activity of the tTAV system with dietary tetracycline and compared gene expression between on and off states. Although each of the conditions and strains tested had a similar number of expressed genes (Table 3 and Fig. 3), we identified 1–3 orders of magnitude difference between the numbers differentially expressed genes in larvae versus those in the adults of the same line (Table 4 and Fig. 4).

In the non-tTAV strain, we failed to detect any differentially expressed genes with a 10% FDR (Table 4). This is surprising, as several authors have reported that in *D. melanogaster* tetracycline disrupts mitochondrial function and leads to trans-generational effects [26, 27]. It has been shown that tetracycline can disrupt mitochondrial protein translation [28], which results in mitonuclear protein imbalance and inhibition of respiration [29]. Respiration inhibition in HEK293 cells disappeared after removal of doxycycline [29]. In addition, RT112 cells exposed to doxycycline were found to differentially express 9.5% of all expressed genes [29]. These findings, and numerous others, illustrate the need for caution in drawing biological conclusions when tetracycline is used for research purposes [30]. However, unlike the present study, many *D. melanogaster* studies did not subject their fly lines to several generations of growth on tetracycline prior to with-versus-without tetracycline experiments. It is possible that by this process we are removing much of the tetracycline-induced variability by selective killing of the *D. melanogaster* microbiota [28], or via mitochondrial damage that takes time to return to normal. Regardless of the reason for this muted response to tetracycline, it appears that any differential expression in our experiments is the result of tTAV activation and not to the use of tetracycline.

By examining genes that are differentially expressed in each insertion line, there may be the expectation that the lethal tTAV phenotype is due to change in expression of critical genes shared between the lines, or that a synthetic positive feedback loop has a standard transcriptional response. However, only larval samples showed genes that were differentially expressed for all insertion sites. Of the 31 differentially expressed genes only four had the same direction of differential expression and the fold change was, generally, extremely low (Table 5). In addition, regression analysis of differentially expressed genes in each strain found that all correlations were significant, but varied in direction (Fig. 5). It appears that not only does the insertion of tTAV have a limited effect on the transcriptome, but also any effect it does have is insertion site-specific with little overlap between sites.

It is possible that the four genes with similar differential expression in larval samples represent a group of core genes responsible for lethality. Two of these, *crok* and *olf*186-*F*, result in recessive lethality when mutated. At first glance, it might seem reasonable that tTAV lethality could be related to changes in expression of these genes, but this does not appear to be true. Although the differential expression is in the same direction for each of the four genes, the magnitudes of the changes are modest, and they differ between insertion lines (Table 5). Both recessive p-element insertions and RNAi knockdowns exist for *crok* and *olf*186-*F*, and both produce lethality as late as the pupa stage [31, 32]. This is in contrast to larval lethality seen in second to third instar stages for each tTAV line. This variation in phenotype and high variability in expression between insertion sites means *crok* and *olf*186-*F* do not offer a simple explanation for *D. melanogaster* lethality.

Another possible reason for tTAV lethality is that the feedback circuit overloads translational or protein catabolism machinery, but our data did not support this conclusion. We searched for previously identified transcriptional markers of translational stress to determine whether this is a viable hypothesis [33]. We checked whether *Xbp*1 (FBgn0021872), *crc* (FBgn0000370), CG7140 (FBgn0037147), *CalX* (FBgn0013995), *MGST*1 (FBgn0025814), *CBS* (FBgn0031148) and *KrT*95*D* (FBgn0020647) are among the differentially expressed genes in any of our strains or developmental stages at FDR = 10%. We only detected differential expression of *crc*, *MGST*1 and CG7140 in the adult 102D–tTAV strain; *crc* was also present in larval 102D–tTAV, 76A2-tTAV and 51D–tTAV, while *MGST*1 was present in larval 76A2-tTAV.

Cellular stresses are known to trigger specific pathway responses and by analysing differentially expressed pathways we sought to capture transcriptome responses shared across all insertion lines that might have been missed in single gene analyses. However, once again, our data did not reveal a clear set of cellular processes represented in all cell types. When GO analysis was performed on the 31 genes differentially expressed in all larval tTAV samples, ubiquitin protease/hydrolase categories were over-represented. Another hypothesis for tTAV lethality is that the circuit overloads the ubiquitin-tagged proteolysis pathway [1] and while the above observation might have been indicative of that, once again, it is unlikely to be the case. As mentioned above, only four of the 31 genes shared the same direction of differential expression, whereas one might have expected a general up-regulation of these pathways in response to overproduction of tTAV protein.

We also explored the hypothesis that activation of the tTAV circuit influences expression in specific genomic regions, either haphazardly or physically near the insertion site, but no evidence of a response was detected.

Although the tTAV system alters gene expression, depending on the integration site, there were no obvious localized effects of the integration. One might assume that the presence of a regulatory region with several activators bound, as is the case with an active tTAV, would potentially influence expression of neighbouring genes. However, after examining gene expression around integration sites we found no evidence of localised tTAV-induced differential expression.

Finally, tTAV protein may be toxic due to the behaviour of either the tetracycline-binding domain, tetR, or the activator domain, VP16. Early in vitro work on a fusion between the DNA binding domain of yeast GAL4 protein and VP16 demonstrated that this construct inhibited transcription of promoters lacking GAL4-binding domains [34–36]. In vivo work in *Saccharomyces cerevisiae* showed a link between transcriptional squelching of a reporter construct and dosage of VP16 [37]. This work also demonstrated that in addition to activation activity, VP16, the DNA-binding domain of the fusion protein was required for squelching. To check for a squelching phenotype, we examined differentially expressed genes for a general tendency toward down-regulation when the tTAV system was active. However we failed to detect a clear pattern of squelching, which strongly suggests that this squelching behaviour is likely not the cause of tTAV lethality.

Although we didn’t detect transcriptional depression, there could potentially be other mechanisms, or else our system is not sensitive enough to detect transient squelching. Several versions of VP16 have been developed that reduce activity and are tolerated at higher levels in HELA cells [38]. Additional study of these modified VP16 elements in our *D. melanogaster* system could further dissect potential squelching. Finally, it may also be worth investigating the behaviour of these modified VP16 elements in insect control systems that do not rely on tTAV positive feedback [7, 14, 15].

There are several reasons why it is difficult to categorically refute the proposed mechanisms of tTAV lethality. Our time point late in the second instar may fail to capture the precise event leading to death. Also it may be that lethality occurs due to a translational level event or is due to post-translational modification; hence our transcriptome analysis would miss these events. It may also be that lethality is related to highly tissue-specific expression. However, it is clear that tTAV activation has a large impact on the transcriptome and that this impact is specific to the tTAV genome integration locus. Given that there is very little overlap between the transcript profiles of each integration site and that there is no evidence for alternative explanations, we suggest that tTAV lethality is the result of integration site-based stochastic differential gene expression, perhaps due to tissue-specific expression patterns. Next, temporal and spatial expression patterns can be explored using specific promoters, such as those relating to the UAS/GAL4 system and alternative feedback systems. This will further illuminate the basis of tTAV lethality and will characterize cellular effects of transcriptional positive feedback.

## Conclusion

Although variation due to genome insertion position is a well-characterised phenomenon, discussion has tended to focus on expression variability of the inserted construct and not on the entire transcriptome. This can be further complicated when the inserted construct is a circuit that can be activated. Here we show that the tTAV system, when activated, influences the whole transcriptome but that these expressions differences are unpredictable. Specifically, there was very little overlap in expression between any of the insertion sites. Furthermore, there was no discernible pattern in the types of transcripts affected, as assessed with GO analysis, nor were there any common genomic regions affected. Finally, expression differences within each strain did not appear to be localised to the insertion site. Our data suggest that the hypothesis that the lethality is caused by a direct effect on transcription of a set of key genes or pathways may be incorrect. Rather than a specific action of a tTAV protein, it is the stochastic transcriptional effects specific to each insertion site that contribute to the tTAV-induced mortality.

It is imperative to develop and characterize disease vector and crop pest control systems that are effective, targeted, consistent and cost-effective. Identifying transcriptomic underpinnings of cryptic lethality phenotypes can improve and refine the technology, helping it to gain public trust [39]. In addition, regulated systems, like tTAV, provide researchers with powerful tools to distinguish cis and trans effects in gene regulation at the whole transcriptome level.

## Methods

### Development of transformation plasmid

The tTAV system was synthesised using DNA 2.0, based on descriptions of the RIDL system [1–3]. This fragment was subsequently cloned into the multiple cloning site of *pattB* [40] and verified by sequencing. The full sequence of the plasmid is provided at 10.6084/m9.figshare.5700958.v1.

### Fly strains

Strains of flies used in experiments described here can be found in Table 6. Transgenic flies were created by injection of the tTAV construct into previously described docking lines that represented a cross section of the *D. melanogaster* genome [40, 41]. Importantly, with respect to the difficulties of obtaining tTAV homozygous lines, the docking lines previously described are all homozygous viable. Potential transformants are screened for red eyes indicating complementation of the *w* ^*1118*^ from the *w* ^*+mc*^ marker present on the tTAV construct. All fly injections and transformant screening was performed by BestGene, Chino Hills, USA.

**Table 6:** Fly stocks used

Strain ID	Genotype	Origin
19E7-tTAV	y^1^ w^1118^ PBac{y + -attP-9A, tTAV}VK00006	Bloomington DSC line 9726 injected with tTAV construct
22A–tTAV	y^1^ M{vas-int.Dm}ZH-2A w*; M{3xP3-RFP.attP,tTAV}ZH-22A	Bloomington DSC line 24,481 injected with tTAV construct
28E7-tTAV	y^1^ w^1118^; PBac{y^+^-attP-3B, tTAV}VK00002	Bloomington DSC line 9723 injected with tTAV construct
43A1-tTAV	y^1^ w^1118^; PBac{y^+^-attP-9A, tTAV}VK00014	Bloomington DSC line 9733 injected with tTAV construct
51C–tTAV	y^1^ M{vas-int.Dm}ZH-2A w*; M{3xP3-RFP.attP, tTAV}ZH-51C	Bloomington DSC line 24,482 injected with tTAV construct
51D–tTAV	y^1^ M{vas-int.Dm}ZH-2A w*; M{3xP3-RFP.attP, w^+mc^ *,*tTAV}ZH-51D/CyO	Bloomginton DSC line 24,483 injected with tTAV construct
68E–tTAV	y^1^ M{vas-int.Dm}ZH-2A w*; M{3xP3-RFP.attP, tTAV}ZH-68E	Bloomington DSC line 24,485 injected with tTAV construct
76A2-tTAV	y^1^ w^1118^; PBac{y^+^-attP-9A, w[+mc]*,* tTAV}VK00013/TM1	Bloomington line 9732 injected with tTAV construct
86Fb-tTAV	y^1^ M{vas-int.Dm}ZH-2A w*; M{3xP3-RFP.attP, w^+mc^ *,*tTAV}ZH-86Fb/TM1	Bloomington DSC line 24,749 injected with tTAV construct
102D–tTAV	y^1^ M{vas-int.Dm}ZH-2A w*; M{3xP3-RFP.attP, w^+mc^ *,*tTAV}ZH-102D	Bloomington DSC line 24,488 injected with tTAV construct
Y-hid	w^1118^/ hs-hid	VDRC line 60,001
Ploen	Wild Caught in Plön, Germany	Isofemale line generated in the Reed group and grown for 10+ generations in the lab.
White Eyed	w^1118^/ hs-hid	Cross between male Y-hid and female Ploen flies selecting w^1118^ individuals

### Fly rearing and media

Flies were incubated on standard Bloomington media at 24 °C under a 14 h light / 10 h dark cycle in either 50 mL vials or 300 mL bottles. Tetracycline media for stock maintenance was made by adding an appropriate volume of 100 mg/mL of tetracycline suspended in 99% ethanol to the surface of solid prepared food.

For survival tests, tetracycline media was made by adding an appropriate amount of antibiotic to achieve a final concentration of 100, 10 or 1 μg/mL and by diluting with unsupplemented media to the required concentration.

For microarray experiments, TET-On media was made by adding tetracycline to a final concentration of 100 μg/mL to cooled (>65 °C) media. Approximately 10 mL of this was added to 50 mL vials. TET-Off media was the same batch of food prior being supplemented with tetracycline.

### tTAV survival tests

The tTAV circuit contains the red eye marker, *w* ^*+mc*^ *,* which allows it to be tracked when crossed with white eyed, *w* ^*1118*^ *,* virgins. A two-generation crossing system was used in all survival experiments. To aid collection of virgin females, a wild stock (‘Plön’) was crossed to ‘Y-hid’, which allows killing of male larvae via incubation at 37 °C for 30 min. Since tTAV is marked with a functional copy of the *white* gene, conferring red eyes, all individuals in the first generation were red-eyed and contained one copy of the tTAV circuit. Three males from this first generation were then backcrossed to 15 females from the ‘White Eyed’ line in 50-mL vials. This particular crossing regime was employed such that the second generation contained 50% tTAV / wild flies; thus, even when conditions did not permit survival of many tTAV flies, there were enough individuals to permit survival of the vial. Parental flies were removed after 5 days. Starting the day after the first offspring emerged, they were counted. Survivability is determined by counting both red- and white-eyed flies, adding these totals for each vial, and dividing the red-eyed files by the total. Each data point was derived from 10 biological replicates, but vials that failed produce any flies were removed from the analysis. Plots in Fig. 2 were made with *ggplot2* [42].

### Microarray flies and RNA extraction

The tTAV lines and non-tTAV control were maintained on TET-on media for 5 generations prior to commencing. Thirty flies, 15 male and 15 female, of the same age, from each of the strains in Table 2, were transferred to either TET-On or TET-Off media. Five days after transfer, adult flies were removed and 10 adult flies, 5 male & 5 female, were frozen at −80 °C. Ten larvae from each of these matings were collected at the late second instar stage, the last life stage normally seen prior to lethality, and frozen at −80 °C. Three biological replicates were produced for each combination of strain, life-stage, and media. RNA was prepared from frozen samples using Trizol and DNAse was treated using a PureLink RNA Mini Kit (Invitrogen).

### Generating microarray data

**Fig. 6:**
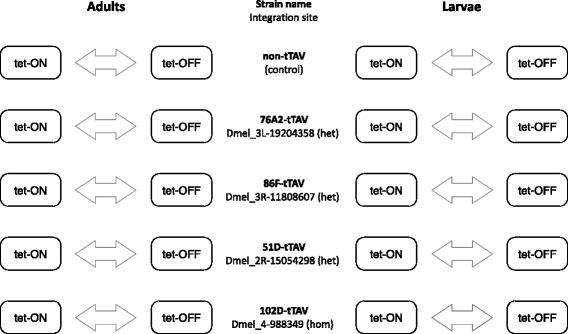
Diagram of experimental design. Two life stages (adults and larve) were tested in each of the 5 transgenic lines, 4 of them harbouring heterozygous integration of the tTAV constructs (het) and one homozygous (hom). All differential gene expression was assessed between tetracycline ON (tet-ON) and tetracycline OFF (tet-OFF) conditions (double-ended arrows) only within each single strain and life stage to ensure that observed effects are due to different treatments and not different genetic backgrounds

Microarray experiments employed Agilent *Drosophila* Gene Expression Microarrays 4x44K and were scanned on an Agilent G2505C scanner (Agilent Technologies). Data collection was divided into two separate experiments. A pilot experiment for strain 102D consisting of two life stages in two conditions each with three biological replicates – a total of 12 samples. The remaining four strains were run as a separate experiment with a total of 48 samples. For each experiment, sample chip position was randomized to avoid genotype- and treatment-specific batch effects. Fig. 6 schematically shows the experimental design.

### Normalisation of microarray data

Microarray data were normalized using the *limma* package [43] in R [44]. Specifically, the microarray background was corrected using the *limma normexp* function and data were normalized with *limma quantile* separately for each life stage. Following normalization, low intensity and control probes were removed as per the *limma* user guide (rev. 9 June 2015). Low intensity was defined as having at least 10% lower intensity than the 95th percentile of negative control probes on at least 3 arrays in each stage. The principal component analysis (*stats* package in R) was used to check for unusual groupings of samples that could indicate confounding effects and relative log expression analysis was used to check for sample outliers with regard to expression level distribution [45] (Additional File 4: Figure S1 and Additional file 5: Figure S2).

### Annotation of microarray data

Probes were annotated using MEGABLAST [46]. All probe names and sequences were extracted from the array, and a total of 32,162 unique probes were used as input in a local MEGABLAST (ver. 2.2.31+) against the Ensembl *Drosophila* cDNA database of all known and predicted genes (downloaded on 15th January 2016). MEGABLAST settings used were:

blastn -task megablast -db db.fa -query query.txt -dust no -max_target_seqs 1 -outfmt “6 qseqid sseqid evalue pident stitle”.

Hits with 100% sequence identity, 92.3% of all 26,665 unique hits, and an e-value ≤1e-20 were retained (total 23,752) and Ensembl Gene IDs were extracted from the retained hit description field. Positional information for each gene was obtained using the BioMart database with the R package *biomaRt* [47]. Comparisons between our annotation and that of the manufacturer revealed that there were 5108 (22%) “mismatched” probes in our set. However, when we compared the overlap of probes after normalization and low intensity filtering, the “mismatched” probe fraction was reduced to at most 12%, indicating that almost half of the probes removed during filtering were those for which the manufacturer’s annotation differed from ours.

All further analyses were done on our set of MEGABLAST–annotated probes. After normalization and low intensity filtering, each sample was filtered to contain only probes present in our set.

### Differentially expressed gene analysis

A moderated t test [43] was used to compare tet-on vs tet-off expression levels for each probe within each life stage and strain only, ie. the differentially expressed genes were all identified by comparing a single strain and life stage of transgenic flies in two different states: with tetracycline and without tetracycline. Therefore, any effects on transcriptome detected could only be due to the dietary tetracycline and not due to differences in genetic background. We used Benjamin-Hofberg correction for multiple testing using the *limma package* [43]. FDR thresholds at 1%, 5%, 10%, 15% and 25% were tested and produced similar overall pattern of gene expression differences, but 10% FDR was the lowest FDR tested for which we obtained large enough numbers of differentially expressed genes to enable analysis of GO categories and genome-wide positional effects and avoided potential false positives such as differentially expressed genes in non-tTAV strain (see Table S3 in Additional File 6).

Ensuring that we used a single probe per Ensembl Gene ID in the dataset further reduced the complexity. Multiple copies of identical probes with *p* values both under and over the threshold of 0.05 (moderated t-test) were removed completely. In cases where there were multiple probes with multiple p values assigned to a single Ensembl Gene ID, we retained only the probe with the lowest p value.

Common gene sets diagrams, principal component analysis, and genomic plots were generated using *limma*, *UpSetR* [48] *prcomp* and Bioconductor [47] packages. Sliding window analysis was run using a custom function. Plots and diagrams were made with *ggplot2* [42].

## Additional files

Additional file 1: Table S1. Gene ontology analysis of differentially expressed genes in transgenic strains. (DOCX 27 kb)

Additional file 2: Table S2. Gene ontology analysis of 31 differentially expressed genes shared among all transgenic strains in larvae. (DOCX 15 kb)

Additional file 3: Figure S3. Proportion of differentially expressed genes (with vs without tetracycline) in 10-gene windows across genes commonly expressed in all adult (A, *n* = 9538) and larvae (B, *n* = 7773) strains. (DOCX 10589 kb)

Additional file 4: Figure S1. Principal component analysis for adults and larvae from all strains. (DOCX 124 kb)

Additional file 5: Figure S2. Relative log expression plots for adults and larvae from all heterozygous strains. (DOCX 1671 kb)

Additional file 6: Table S3. Number of differentially expressed genes at different levels of false discovery rate for all strains and stages. (DOCX 67 kb)

## Abbreviations

FDR: False discovery rate
GO: Gene ontology
SIT: Sterile insect treatment
tTAV: Tetracycline-controlled transactivator
