# Supplementary material for: Transcriptional effects of a positive feedback circuit in Drosophila melanogaster
Source: BMC Genomics. 2017 Dec 28;18:990. doi: 10.1186/s12864-017-4385-z (PMC5746007; doi:10.1186/s12864-017-4385-z)
Supplement: Supplementary file 1 — Gene ontology analysis of differentially expressed genes in transgenic strains. (DOCX 27 kb) [file 12864_2017_4385_MOESM1_ESM.docx]

**Table S1. Gene ontology analysis of differentially expressed genes in transgenic strains.** Categories common to more than one strain are highlighted in yellow; there are no categories common to all transgenic strains. Only p values < 0.01 are shown. Ont: ontology categories: CC: cellular component; BP: biological process; MF: molecular function; N: number of genes observed in a etegory; DE: number of genes expected in a category; P.DE: p value.

| Strain | GO ID | Term | Ont | N | DE | P.DE |
| --- | --- | --- | --- | --- | --- | --- |
| A.S001 | GO:0044429 | mitochondrial part | CC | 358 | 111 | 0.0002 |
| A.S001 | GO:0005739 | mitochondrion | CC | 539 | 157 | 0.0004 |
| A.S001 | GO:0030120 | vesicle coat | CC | 23 | 13 | 0.0005 |
| A.S001 | GO:0016616 | oxidoreductase activity, acting on the CH-OH group of donors, NAD or NADP as acceptor | MF | 65 | 27 | 0.0007 |
| A.S001 | GO:0030125 | clathrin vesicle coat | CC | 9 | 7 | 0.0008 |
| A.S001 | GO:0016491 | oxidoreductase activity | MF | 518 | 149 | 0.0010 |
| A.S001 | GO:0030662 | coated vesicle membrane | CC | 25 | 13 | 0.0015 |
| A.S001 | GO:0016627 | oxidoreductase activity, acting on the CH-CH group of donors | MF | 34 | 16 | 0.0017 |
| A.S001 | GO:0030665 | clathrin-coated vesicle membrane | CC | 10 | 7 | 0.0021 |
| A.S001 | GO:0030594 | neurotransmitter receptor activity | MF | 23 | 12 | 0.0022 |
| A.S001 | GO:0030659 | cytoplasmic vesicle membrane | CC | 26 | 13 | 0.0024 |
| A.S001 | GO:0030132 | clathrin coat of coated pit | CC | 6 | 5 | 0.0031 |
| A.S001 | GO:0030130 | clathrin coat of trans-Golgi network vesicle | CC | 6 | 5 | 0.0031 |
| A.S001 | GO:0012510 | trans-Golgi network transport vesicle membrane | CC | 6 | 5 | 0.0031 |
| A.S001 | GO:0030658 | transport vesicle membrane | CC | 13 | 8 | 0.0032 |
| A.S001 | GO:0006900 | membrane budding | BP | 24 | 12 | 0.0035 |
| A.S001 | GO:0048037 | cofactor binding | MF | 149 | 49 | 0.0036 |
| A.S001 | GO:0055114 | oxidation-reduction process | BP | 467 | 132 | 0.0039 |
| A.S001 | GO:0031427 | response to methotrexate | BP | 11 | 7 | 0.0046 |
| A.S001 | GO:0012506 | vesicle membrane | CC | 31 | 14 | 0.0053 |
| L.S001 | GO:0003384 | apical constriction involved in gastrulation | BP | 7 | 4 | 0.0000 |
| L.S001 | GO:0003381 | epithelial cell morphogenesis involved in gastrulation | BP | 7 | 4 | 0.0000 |
| L.S001 | GO:0003383 | apical constriction | BP | 16 | 4 | 0.0014 |
| L.S001 | GO:0009062 | fatty acid catabolic process | BP | 28 | 5 | 0.0018 |
| L.S001 | GO:0006631 | fatty acid metabolic process | BP | 72 | 8 | 0.0021 |
| L.S001 | GO:0070252 | actin-mediated cell contraction | BP | 18 | 4 | 0.0022 |
| L.S001 | GO:0072329 | monocarboxylic acid catabolic process | BP | 30 | 5 | 0.0024 |
| L.S001 | GO:0044242 | cellular lipid catabolic process | BP | 44 | 6 | 0.0026 |
| L.S001 | GO:0016408 | C-acyltransferase activity | MF | 10 | 3 | 0.0033 |
| L.S001 | GO:0006635 | fatty acid beta-oxidation | BP | 21 | 4 | 0.0040 |
| L.S001 | GO:0003382 | epithelial cell morphogenesis | BP | 22 | 4 | 0.0048 |
| L.S001 | GO:0030048 | actin filament-based movement | BP | 24 | 4 | 0.0067 |
| L.S001 | GO:0030307 | positive regulation of cell growth | BP | 24 | 4 | 0.0067 |
| L.S001 | GO:0016579 | protein deubiquitination | BP | 38 | 5 | 0.0069 |
| L.S008 | GO:0035262 | gonad morphogenesis | BP | 6 | 6 | 0.0002 |
| L.S008 | GO:0016410 | N-acyltransferase activity | MF | 46 | 22 | 0.0004 |
| L.S008 | GO:0016407 | acetyltransferase activity | MF | 48 | 22 | 0.0008 |
| L.S008 | GO:0007097 | nuclear migration | BP | 18 | 11 | 0.0009 |
| L.S008 | GO:0045450 | bicoid mRNA localization | BP | 9 | 7 | 0.0010 |
| L.S008 | GO:0005811 | lipid particle | CC | 184 | 63 | 0.0010 |
| L.S008 | GO:0008080 | N-acetyltransferase activity | MF | 43 | 20 | 0.0010 |
| L.S008 | GO:0006458 | 'de novo' protein folding | BP | 7 | 6 | 0.0011 |
| L.S008 | GO:0005851 | eukaryotic translation initiation factor 2B complex | CC | 7 | 6 | 0.0011 |
| L.S008 | GO:0040023 | establishment of nucleus localization | BP | 21 | 12 | 0.0012 |
| L.S008 | GO:0044429 | mitochondrial part | CC | 300 | 95 | 0.0013 |
| L.S008 | GO:0051647 | nucleus localization | BP | 27 | 14 | 0.0016 |
| L.S008 | GO:0007189 | adenylate cyclase-activating G-protein coupled receptor signaling pathway | BP | 8 | 6 | 0.0034 |
| L.S008 | GO:0048567 | ectodermal digestive tract morphogenesis | BP | 6 | 5 | 0.0038 |
| L.S008 | GO:0016679 | oxidoreductase activity, acting on diphenols and related substances as donors | MF | 13 | 8 | 0.0043 |
| L.S008 | GO:0043168 | anion binding | MF | 809 | 225 | 0.0050 |
| L.S008 | GO:0031975 | envelope | CC | 262 | 81 | 0.0059 |
| L.S008 | GO:0010324 | membrane invagination | BP | 11 | 7 | 0.0060 |
| L.S008 | GO:0006911 | phagocytosis, engulfment | BP | 11 | 7 | 0.0060 |
| L.S011 | GO:0031386 | protein tag | MF | 7 | 3 | 0.0001 |
| L.S011 | GO:0050770 | regulation of axonogenesis | BP | 30 | 4 | 0.0004 |
| L.S011 | GO:0010769 | regulation of cell morphogenesis involved in differentiation | BP | 56 | 5 | 0.0006 |
| L.S011 | GO:0010975 | regulation of neuron projection development | BP | 61 | 5 | 0.0008 |
| L.S011 | GO:0031344 | regulation of cell projection organization | BP | 84 | 5 | 0.0035 |
| L.S011 | GO:0035371 | microtubule plus-end | CC | 8 | 2 | 0.0039 |
| L.S011 | GO:0045664 | regulation of neuron differentiation | BP | 89 | 5 | 0.0045 |
| L.S011 | GO:0009126 | purine nucleoside monophosphate metabolic process | BP | 96 | 5 | 0.0062 |
| L.S011 | GO:0009167 | purine ribonucleoside monophosphate metabolic process | BP | 96 | 5 | 0.0062 |
| L.S011 | GO:1902667 | regulation of axon guidance | BP | 10 | 2 | 0.0062 |
| L.S011 | GO:0005881 | cytoplasmic microtubule | CC | 11 | 2 | 0.0075 |
| L.S011 | GO:0008227 | G-protein coupled amine receptor activity | MF | 11 | 2 | 0.0075 |
| L.S011 | GO:0006123 | mitochondrial electron transport, cytochrome c to oxygen | BP | 11 | 2 | 0.0075 |
| L.S011 | GO:0050920 | regulation of chemotaxis | BP | 11 | 2 | 0.0075 |
| L.S011 | GO:0044248 | cellular catabolic process | BP | 447 | 12 | 0.0078 |
| L.S011 | GO:0009123 | nucleoside monophosphate metabolic process | BP | 102 | 5 | 0.0080 |
| L.S011 | GO:0009161 | ribonucleoside monophosphate metabolic process | BP | 102 | 5 | 0.0080 |
| L.S011 | GO:0044282 | small molecule catabolic process | BP | 68 | 4 | 0.0093 |
| L.S206 | GO:0016579 | protein deubiquitination | BP | 36 | 6 | 0.0020 |
| L.S206 | GO:0006631 | fatty acid metabolic process | BP | 65 | 8 | 0.0028 |
| L.S206 | GO:0001085 | RNA polymerase II transcription factor binding | MF | 17 | 4 | 0.0031 |
| L.S206 | GO:0044242 | cellular lipid catabolic process | BP | 40 | 6 | 0.0034 |
| L.S206 | GO:0044255 | cellular lipid metabolic process | BP | 204 | 16 | 0.0041 |
| L.S206 | GO:0006635 | fatty acid beta-oxidation | BP | 19 | 4 | 0.0048 |
| L.S206 | GO:0016408 | C-acyltransferase activity | MF | 10 | 3 | 0.0051 |
| L.S206 | GO:0016747 | transferase activity, transferring acyl groups other than amino-acyl groups | MF | 121 | 11 | 0.0055 |
| L.S206 | GO:0004843 | ubiquitin-specific protease activity | MF | 34 | 5 | 0.0081 |
| L.S206 | GO:0036459 | ubiquitinyl hydrolase activity | MF | 34 | 5 | 0.0081 |
| L.S206 | GO:0044432 | endoplasmic reticulum part | CC | 95 | 9 | 0.0090 |
| L.S206 | GO:0070646 | protein modification by small protein removal | BP | 49 | 6 | 0.0095 |
