# Supplementary material for: Transcriptional effects of a positive feedback circuit in Drosophila melanogaster
Source: BMC Genomics. 2017 Dec 28;18:990. doi: 10.1186/s12864-017-4385-z (PMC5746007; doi:10.1186/s12864-017-4385-z)
Supplement: Supplementary file 2 — Gene ontology analysis of 31 differentially expressed genes shared among all transgenic strains in larvae. (DOCX 15 kb) [file 12864_2017_4385_MOESM2_ESM.docx]

**Table S2. Gene ontology analysis of 31 differentially expressed genes shared among all transgenic strains in larvae.**

Categories are ordered by increasing p value. Ont: ontology categories: CC: cellular component; BP: biological process; MF: molecular function; N: number of genes observed in a etegory; DE: number of genes expected in a category; P.DE: p value.

| GO ID | Term | Ont | N | DE | P.DE |
| --- | --- | --- | --- | --- | --- |
| GO:0051908 | double-stranded DNA 5'-3' exodeoxyribonuclease activity | MF | 1 | 1 | 0.0043 |
| GO:0008852 | exodeoxyribonuclease I activity | MF | 1 | 1 | 0.0043 |
| GO:0048256 | flap endonuclease activity | MF | 1 | 1 | 0.0043 |
| GO:0035236 | proctolin receptor activity | MF | 1 | 1 | 0.0043 |
| GO:0045145 | single-stranded DNA 5'-3' exodeoxyribonuclease activity | MF | 1 | 1 | 0.0043 |
| GO:0004843 | thiol-dependent ubiquitin-specific protease activity | MF | 32 | 2 | 0.0081 |
| GO:0036459 | thiol-dependent ubiquitinyl hydrolase activity | MF | 32 | 2 | 0.0081 |
| GO:0101005 | ubiquitinyl hydrolase activity | MF | 32 | 2 | 0.0081 |
| GO:0035312 | 5'-3' exodeoxyribonuclease activity | MF | 2 | 1 | 0.0086 |
| GO:0034274 | Atg12-Atg5-Atg16 complex | CC | 2 | 1 | 0.0086 |
| GO:0004167 | dopachrome isomerase activity | MF | 2 | 1 | 0.0086 |
| GO:0016320 | endoplasmic reticulum membrane fusion | BP | 2 | 1 | 0.0086 |
| GO:0052716 | hydroquinone:oxygen oxidoreductase activity | MF | 2 | 1 | 0.0086 |
| GO:0006583 | melanin biosynthetic process from tyrosine | BP | 2 | 1 | 0.0086 |
