# Supplementary material for: Transcriptional effects of a positive feedback circuit in Drosophila melanogaster
Source: BMC Genomics. 2017 Dec 28;18:990. doi: 10.1186/s12864-017-4385-z (PMC5746007; doi:10.1186/s12864-017-4385-z)
Supplement: Supplementary file 3 — Proportion of differentially expressed genes (with vs without tetracycline) in 10-gene windows across genes commonly expressed in all adult (A, n = 9538) and larvae (B, n = 7773) strains. (DOCX 10589 kb) [file 12864_2017_4385_MOESM3_ESM.docx]

**Figure S3. Proportion of differentially expressed genes (with vs without tetracycline) in 10-gene windows across genes commonly expressed in all adult (A, n=9538) and larvae (B, n=7773) strains.**

Results of the sliding window analysis. We constructed gene sets with 9538 and 7773 genes expressed in all strains in aduls and larvae, respectively and ordered them according to their genomic positions. We then took 10 neighbouring genes at a time in windows overlapping by 9 genes and asked how many of the genes in each window are differentially expressed. This proportion is plotted for every window in every strain and life stage. If the differentially expressed genes were clustered in a genomic region, we should observe distinct peaks of windows with high proportion of differentially expressed genes in them.

**A**

**B**
