# Supplementary material for: Transcriptional effects of a positive feedback circuit in Drosophila melanogaster
Source: BMC Genomics. 2017 Dec 28;18:990. doi: 10.1186/s12864-017-4385-z (PMC5746007; doi:10.1186/s12864-017-4385-z)
Supplement: Supplementary file 4 — Principal component analysis for adults and larvae from all strains. (DOCX 124 kb) [file 12864_2017_4385_MOESM4_ESM.docx]

**Figure S1 Principal component analysis for adults and larvae from all strains**

Principal component analysis was run on all genes expressed in each strain and life stage. For all larvae strains except non-tTAV and homozygous 102D-tTAV we observe clear separation of the samples based on treatment (with vs without tetracycline, tet ON and tet OFF, respectively). For adults, all but the homozygous and 51D-tTAV strains show no differentiation based on treatment.
