# Supplementary material for: Transcriptional effects of a positive feedback circuit in Drosophila melanogaster
Source: BMC Genomics. 2017 Dec 28;18:990. doi: 10.1186/s12864-017-4385-z (PMC5746007; doi:10.1186/s12864-017-4385-z)
Supplement: Supplementary file 5 — Relative log expression plots for adults and larvae from all heterozygous strains. (DOCX 1671 kb) [file 12864_2017_4385_MOESM5_ESM.docx]

**Figure S2 Relative log expression plots for adults and larvae from all heterozygous strains**

Relative log expression (RLE) plots demonstrating good quality of the signal across all samples. RLE is calculated as the difference between the spot intensity and the median intensity for the same feature across all the arrays. Each boxplot is a sample; for a high quality of the signal, the boxplots should be centred around zero and have approximately the same dispersion.
