# Supplementary material for: Transcriptional effects of a positive feedback circuit in Drosophila melanogaster
Source: BMC Genomics. 2017 Dec 28;18:990. doi: 10.1186/s12864-017-4385-z (PMC5746007; doi:10.1186/s12864-017-4385-z)
Supplement: Supplementary file 6 — Number of differentially expressed genes at different levels of false discovery rate for all strains and stages. (DOCX 67 kb) [file 12864_2017_4385_MOESM6_ESM.docx]

**Table S3. Number of differentially expressed genes at different levels of false discovery rate for all strains and stages.**

The overall picture of gene expression differences does not change with increasing FDR threshold. We used 10% FDR level in all comparisons as a compromise between number of false positives and number of putatively differentially expressed genes required to perform gene ontology and sliding window analyses. This threshold is more conservative with regard to our finding of the lack of consistent pattern of gene expression differences. A chosen FDR value of 10% is shaded in grey.

| **Adults** |  |  |  |  |  |
| --- | --- | --- | --- | --- | --- |
| **FDR level** | **102D-tTAV** | **Non-tTAV** | **76A2-tTAV** | **86F-tTAV** | **51D-tTAV** |
| 1% | 8 | 0 | 0 | 3 | 1 |
| 5% | 1135 | 0 | 0 | 3 | 1 |
| 10% | 2301 | 0 | 0 | 3 | 1 |
| 15% | 3154 | 0 | 0 | 3 | 1 |
| 25% | 4334 | 0 | 0 | 3 | 3 |
|  |  |  |  |  |  |
| **Larvae** |  |  |  |  |  |
| **FDR level** | **102D-tTAV** | **Non-tTAV** | **76A2-tTAV** | **86F-tTAV** | **51D-tTAV** |
| 1% | 0 | 0 | 32 | 0 | 21 |
| 5% | 98 | 0 | 1271 | 23 | 137 |
| 10% | 311 | 0 | 2116 | 115 | 336 |
| 15% | 954 | 1 | 2775 | 282 | 586 |
| 25% | 1996 | 10 | 3898 | 840 | 1215 |
